# The promotional effect of microRNA-103a-3p in cervical cancer cells by regulating the ubiquitin ligase FBXW7 function

**DOI:** 10.1007/s13577-021-00649-2

**Published:** 2022-01-30

**Authors:** Li Ren, Jinjin Yang, Xiyan Meng, Junjun Zhang, Yiran Zhang

**Affiliations:** Department of Gynaecology, The First Affiliated Hospital, and College of Clinical Medicine of Henan University of Science and Technology, No. 24 Jinghua Road, Jianxi District, Luoyang, 471003 China

**Keywords:** Cervical cancer, MicroRNA-103a-3p, FBXW7, Cell proliferation, Cell apoptosis

## Abstract

MicroRNAs (miRNAs) have been reported to be involved in the initiation and progression of human tumors including cervical cancer (CC). However, the mechanisms underlying of their actions in CC remain to be fully elucidated. Herein, the differentially expressed miRNAs that were screened based on GSE55940 microarray data retrieved from Gene Expression Omnibus (GEO), and miR-103a-3p was significantly upregulated in CC tissues which was selected as the target miRNA for further research. We also found that high expression of miR-103a-3p was closely associated with histological grades, FIGO stage and distant metastasis as well as reflected poor overall survival. Moreover, miR-103a-3p inhibition decreased the growth capacity of SiHa and HeLa cells by inducing cell apoptosis. And F-box and WD repeat-domain containing protein 7 (FBXW7), a well-known tumor suppressor in many cancer types, was identified as a direct target of miR-103a-3p. It was further found that FBXW7 was significantly downregulated in CC tissues, and it was inversely correlated with miR-103a-3p expression levels. Further investigation demonstrated that FBXW7 upregulation could simulate the roles of miR-103a-3p knockdown in cell viability and apoptosis. Moreover, FBXW7 knockdown efficiently abrogated the influences of CC cells proliferation caused by miR-103a-3p inhibition. Notably, miR-103a-3p could block FBXW7 mediated the downstream transcription factor pathways. Taken together, these findings suggest that miR-103a-3p functions as an oncogene in CC by targeting FBXW7.

## Introduction

In the last few years, great effort has been made towards the diagnosis and therapeutics of cervical cancer (CC), which firmly ranks the second most common cancer in women worldwide [1]. Even so, about 500,000 women still develop cervical cancer worldwide, and approximately 60% of patients die of the disease each year [2]. Therefore, it is important to screen therapeutic targets for CC treatment.

MicroRNAs (miRNAs), normally containing about 21–25 nucleotides, are a class of small non-coding RNA molecules involved in the regulation of gene expression by targeting mRNAs for translational repression or cleavage [3, 4]. Growing evidence indicates that miRNAs are frequently implicated in many types of human cancers, with significant effects on cellular processes including cell apoptosis, proliferation, and so on [5]. During the tumorigenesis and development of CC, miRNAs have been reported to be oncogenes or tumor suppressor genes [6–8]. For instance, Lv et al. [9] found that miR-664 exerted anti-tumor effects by directly targeting c-Kit in CC. Liao et al. [10] have reported that miR‑874 was significantly downregulated in CC tissues, and inhibited cancer progression by directly targeting E26 transformation specific-1 (ETS1). Additionally, Tan et al. [11] found miR-378 promoted the migratory and invasive abilities of CC cells by directly targeting autophagy-related protein 12. Wei et al. [8] reported that miR-221-3p promoted CC metastasis by directly targeting twist homolog 2 (TWIST2). Additionally, some miRNAs, such as miR-365 and miR-152, have become potential diagnostic biomarkers in patients with CC [12, 13]. Therefore, identifying more miRNAs that are aberrantly expressed in CC and addressed their underlying molecular mechanisms can be beneficial for therapeutic approaches for CC.

In the present study, the expression levels of miR-103a-3p in CC. Then, the actions of miR-103a-3p on the cell viability and apoptosis in CC were analyzed. Furthermore, the regulatory mechanism underlying the effect of miR-103a-3p in the progression of CC was investigated. Our investigations suggested that miR-103a-3p might function as an oncogenic gene in the development of CC, providing a possible target for the diagnosis and therapy of CC.

## Materials and methods

### Clinical specimens

70 pairs of primary cervical cancer (CC) tissues and corresponding adjacent tissues were obtained from Department of Gynaecology, the First Affiliated Hospital, and College of Clinical Medicine of Henan University of Science and Technology between June 2017 and June 2018. The samples were quickly stored at − 80 °C until use. All patient characteristics are presented in Table 1. Written informed consent was obtained from each subject. This study was approved by the Ethical Committee of the First Affiliated Hospital, and College of Clinical Medicine of Henan University of Science and Technology (Ethical approval number: 2017031) and is in accordance with the current version of the Helsinki Declaration.

**Table 1:** Associations between miR-103a-3p and clinicopathological features of patients with cervical cancer

Feature	Total *n* = 70	miR-103a-3p		*p* value
		High	Low	
Age (years)				0.7132
< 50	21	11	10	
≥ 50	49	28	21	
Tumor size (cm)				0.0644
< 4.0	32	14	18	
≥ 4.0	38	25	13	
Histological grades				0.0011**
Well/moderate	40	29	11	
Poor	30	10	20	
FIGO stage				0.0042**
I/II	34	13	21	
III/IV	36	26	10	
Distant metastasis				0.0140*
Yes	48	22	26	
No	22	17	5	
HPV				0.4556
( +)	59	34	25	
( −)	11	5	6	

*p < 0.05; **p < 0.01

### Analysis of GEO database

Microarray dataset was obtained from GEO database (https://www.ncbi.nlm.nih.gov/geo/query/acc.cgi?acc=GSE55940). GEO2R (www.ncbi.nlm.nih.gov/geo/geo2r/), an interactive web tool was applied to compare the samples in two different groups under the same experimental condition. Differentially expressed miRNAs (DE-miRNAs) were then identified based on the fold changes (FCs). Following this, the miRNAs that were significantly differentially expressed were identified by Volcano Plot filtering with fold change values > 2.0 and *p* < 0.05 as the screening conditions. Finally, the heat map of DE-miRNAs was created using a method of hierarchical clustering by GeneSpring GX, version 7.3 (Agilent Technologies, California, United Stages).

### Quantitative real-time PCR analysis

Total RNA was extracted from tissues or cultured cells using TRIzol (Invitrogen, Thermo Fisher Scientific, Inc., Waltham, MA, USA). Reverse transcription of miRNAs was synthesized using the TaqMan™ MicroRNA Reverse Transcription kit (Thermo Fisher Scientific, Inc., Waltham, MA, USA) and miRNAs expression levels were measured using TaqMan™ MicroRNA Assay kit on an ABI7500 sequence detection system (Applied Biosystems, Thermo Fisher Scientific, Inc). For detection of FBXW7 mRNA, total RNA was reverse-transcribed to cDNA using a Reverse Transcription Kit (Takara Bio, Inc., Tokyo, Japan). Then a SYBR Premix Ex Taq II (Takara Bio, Inc., Tokyo, Japan) was used for PCR. The thermocycling conditions were as follows: Incubation at 95 °C for 5 min, followed by 40 cycles of 95 °C for 15 s, 60 °C for 30 s and 72 °C for 30 s. The primer sequences were as follows: miR-103a-3p RT primer: 5′-GTCGTATCCAGTGCAGGGTCCGAGGTATTCGCACTGGATACGACTCATAG-3′, forward: 5′-CGAGCAGCATTGTACAGGG-3′ and reverse: 5′-GCAGGGTCCGAGGTATTC-3′; U6 forward: 5′-GCTTCGGCAGCACATATACTAAAAT-3′ and reverse: 5′-CGCTTCACGAATTTGCGTGTCAT-3′; FBXW7 forward: 5′-TTCACCAACTCTCCTCCCCATT-3′ and reverse: 5′-GCTGAACATGGTACAAGCCCA-3′; GAPDH forward, 5′-GAAGATGGTGATGGGATTTC-3′, and reverse, 5′-GAAGGTGAAGGTCGGAGT-3′. Expression data were uniformly normalized to the expression of U6 and GAPDH, respectively. The relative expression of each gene was calculated using the 2^−∆∆Ct^ method [14]. All reactions were performed in triplicate.

### Cell culture

The cervical cancer cell lines, HeLa, C-33A, SiHa and CasKi, and the normal ectocervical cell lines (Ect1/E6E7) were obtained from Shanghai cell bank of Chinese Academy of Sciences. SiHa, HeLa, C-33A and Ect1/E6E7 cells were cultured in Dulbecco’s Modified Eagle (DMEM, Hyclone, Logan, UT, USA) supplemented with 10% fetal bovine serum (FBS, Sigma-Aldrich, St. Louis, MO, USA), while CasKi cells were cultured in RPMI 1640 (Gibco, Grand Island, NY, USA) containing 10% FBS. All cells were cultured in humidified 37 °C incubator with 5% CO_2_.

### Cell transfection

miR-103a-3p mimics (5′-AGCAGCATTGTACAGGGCTATGA-3′), mimics negative control (NC) (5′-ATAGTGATCAGATGGGCAGCCTA-3′), miR-103a-3p inhibitor (5′-TCATAGCCCTGTACAATGCTGCT-3′), inhibitor NC (5′-TATCCCACGCTAGCTGTCTTGAA-3′) were obtained from Shanghai GenePharma Co., Ltd. pcDNA3.1-FBXW7, pcDNA3.1 vector, si-FBXW7 (5′-GCTCCCTAAAGAGTTGGCACTCTAT-3′) or si-Scramble (5′-GCTATCGAATGAGGTCACCTCCTAT-3′) were synthesized by Guangzhou RiboBio Co, Ltd. (Guangzhou, China). Then, SiHa and Hela cells (4 × 10^5^ cells per well) were seeded in 6-well plates and cultured overnight until reached about 80% confluence, 100 nM miR-103a-3p mimics, mimics NC, miR-103a-3p inhibitor, inhibitor NC, 2 μg pcDNA3.1-FBXW7 plasmid, and 100 ng si-FBXW7 were transfection using the Lipofectamine 3000 reagent (Invitrogen, Thermo Fisher Scientific, Inc., Waltham, MA, USA) according to the manufacturer’s protocol. After incubation for 6 h, regular culture medium was added into each well to terminate reaction at 37 °C. 48 post-transfection, cells were harvested for subsequent experiments.

### Cell proliferation

The cell viability was measured using a Cell Counting Kit-8 (CCK-8) assay (Beyotime, Shanghai, China) according to the manufacturer’s instructions. The cells (5 × 10^4^cells/well) were seeded in 96-well plate with 100 μl DMEM medium supplemented with 10% FBS. After 48 h incubation, 10 μl CCK-8 reagent was added to each well and continuously cultured for another 2 h. Then, the absorbance was read at 450 nm using a microplate reader (Model 680; Bio-Rad Laboratories, Inc.).

### Cell apoptosis

Annexin V/PI apoptosis-detection kit (Nanjing KeyGen Biotech Co., Ltd.) was used to evaluate the apoptosis according to the manufacturer’s protocol. The stained cells were immediately examined using an EPICS XL-MCL FACScan flow cytometer (Beckman Coulter, Inc., Brea, CA, USA) and analyzed using FlowJo 8.7.1 software (Ashland, OR). The results showed healthy viable cells in the lower left quadrant (Q4) on the scatter plot as (FITC-/PI-). The lower right quadrant (Q3) represented the early stage apoptotic cells as (FITC + /PI-). The upper right quadrant (Q2) represented necrotic cells and late stage apoptotic cells as (FITC + /PI +). Apoptotic rate = percentage of early stage apoptotic cells (Q3) + percentage of late stage apoptotic cells (Q2) [15].

### Caspase-3 activity

After 24 h transfection, transfected cells were collected, lysed, and centrifuged for collecting supernatant. The supernatant was analyzed for caspase-3 activity using a Caspase-3 Activity kit (Beyotime Institute of Biotechnology, Shanghai, China) according to the manufacturer’s protocol. The optical density was then detected using a Model 680 microplate reader (Bio-Rad Laboratories, Inc.) at 405 nm.

### Luciferase reporter assay

Luciferase reporters were generated based on the Firefly luciferase expressing vector (pmirGLO; Promega, Madison, WI, USA). The 3’UTR fragment of the human FBXW7 gene and its mutant of the theoretical miR-103a-3p binding site were cloned into the pmirGLO vector to form the reporter vector, named wild type (wt) and mutant type (mut) of FBXW7 3’UTR, respectively. To construct pmirGLO-FBXW7-3’UTR, a partial 3’UTR of the FBXW7 segment of human FBXW7 mRNA containing the putative miR-103a-3p binding sites was amplified and cloned into the vector pmirGLO. Mutations within potential miR-103a-3p binding sites were introduced using Quick Change Site-Direct Mutagenesis Kit (Stratagene, La Jolla, CA, USA). 48 h after co-transfection of miRNA with reporter vector, cells were harvested and assayed with Dual Luciferase Assay (Promega) according to the manufacturer’s protocol. Firefly luciferase activity was normalized to *Renilla* luciferase activity.

### Immunohistochemistry (IHC)

Two paired CC tissues and adjacent tissues were embedded in paraffin and sectioned at 5 µm thickness. Then, these sections were dewaxed in xylene and rehydrated through graded alcohols. Endogenous peroxidase was inactivated by 3% H_2_O_2_ for 15 min at room temperature. Subsequently, sections were blocked with 10% FBS for 30 min, and incubated with FBXW7 antibody (diluted 1:100; cat no. ab128062, Abcam, USA) at 4 °C overnight. The sections were incubated with goat anti-rabbit IgG H&L (HRP) (cat no. ab6721, Abcam, USA) at room temperature for 30 min. Images were photographed using an Olympus BX51 light microscope (magnification, × 200; Olympus Corporation).

### Western blot analysis

Total protein was extracted with RIPA lysis buffer (Beyotime Biotechnology, Shanghai, China) and quantified with a BCA protein assay kit (Beyotime Institute of Biotechnology, Haimen, China). Next, the proteins (30 μg/lane) were separated by 12% SDS-PAGE gels and transferred to PVDF membranes (Millipore, Bedford, MA, USA), and then blocked with 5% skimmed milk for 2 h at 4 °C overnight. Subsequently, the membranes were incubated at 4 °C overnight with primary antibodies as follows: FBXW7 (1:1000, cat no. ab128062, Abcam, USA), cleaved caspase-3 (1:1000; cat. no. 9602, Cell Signaling Technology, Inc Danvers, MA, USA), Bax (1:1000; cat. no. 5023, Cell Signaling Technology, Inc), Bcl-2 (1:1000; cat. no. 3498, Cell Signaling Technology, Inc), c-Myc (1:1000; cat. no. 18583, Cell Signaling Technology, Inc) Notch2 (1:1000; cat. no. 5732, Cell Signaling Technology, Inc), Yap (1:1000; cat. no.14074, Cell Signaling Technology, Inc), cyclin E (1:1000; cat. no. 20808, Cell Signaling Technology, Inc) and β-actin (1:2000, cat. no. 4970, Cell Signaling Technology, Inc) at 4 °C overnight. The membranes were subsequently incubated with horseradish peroxidase-conjugated anti-rabbit IgG secondary antibody (1:2000; cat. no. 7074, Cell Signaling Technology, Inc) for 1 h at room temperature. The protein expressions were detected using an ECL kit (Santa Cruz Biotechnology, Inc.) and the bands were quantified using Image-Pro Plus version 6.0 software (Media Cybernetics, Inc., Rockville, MD, USA).

### Statistical analysis

Statistical calculations were performed using SPSS 13.0 software package (SPSS, Inc.). All data are presented as the mean ± S.D. Differences between two groups were analyzed by unpaired *t* test and continuous data from multiple groups were analyzed using a one-way ANOVA, followed by Tukey’s post hoc test. The correlation between FBXW7 and miR-103a-3p expression was analyzed using Pearson’s correlation coefficient. Survival curves were calculated by the Kaplan–Meier method by the log-rank test. *p* < 0.05 was considered to indicate a statistically significant difference.

## Results

### miR-103a-3p was significantly upregulated in CC tumor tissues and correlates with clinicopathologic parameters

**Fig. 1:**
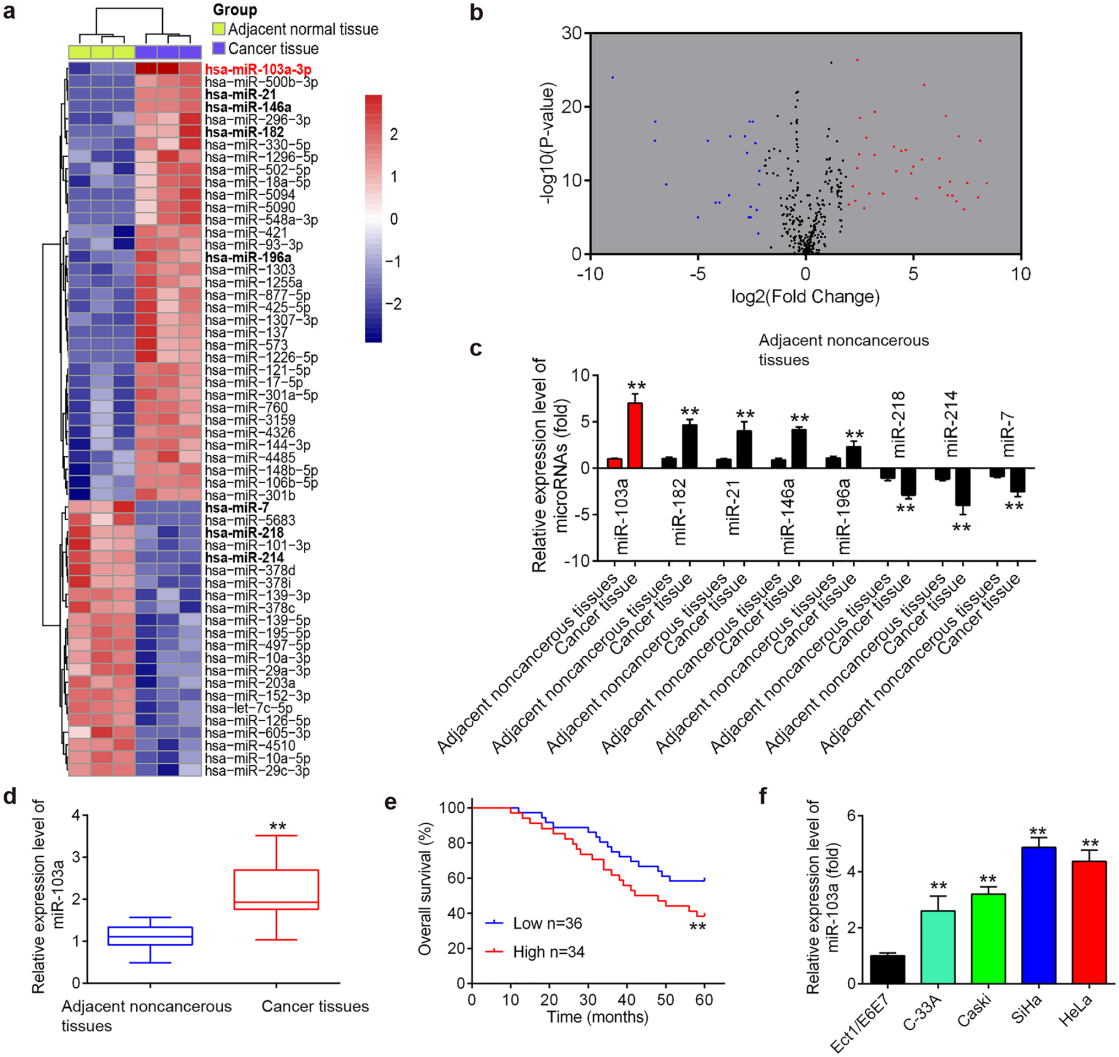
miR-103a-3p was significantly upregulated in CC tissues. **a** Heatmap of normalized expression levels of miRNAs in three pairs of CC tissues and matched tumor-adjacent tissues. Green indicates low expression levels, while red indicates high expression levels. Microarray dataset was obtained from GEO database (https://www.ncbi.nlm.nih.gov/geo/query/acc.cgi?acc=GSE55940). **b** Volcano plot presenting the differentially expressed miRs. *Y*-axis represents log transformed *p* value, and *x*-axis indicates the mean expression differences of miRs between CC tissues and matched tumor-adjacent tissues. |log2FoldChange|> 2 was set as the cut-off criteria. **c** The expression levels of miR-103a, miR-182, miR-21, miR-146a, miR-196a, miR-218, miR-214 and miR-7 were detected in CC tissues and matched tumor-adjacent tissues by qRT-PCR (*n* = 3). Data represent the mean ± SD of three independent experiments. **p < 0.01 vs. adjacent tissues. **d** The expression levels of miR-103a, were detected in CC tissues and matched tumor-adjacent tissues by qRT-PCR (*n* = 70). **e** Kaplan–Meier survival curves of patients with CC according to the expression of miR-103a-3p. **f** The expression levels of miR-103a, were detected in four CC cell lines and normal ectocervical cell lines (Ect1/E6E7) (*n* = 3). Data represent the mean ± SD of three independent experiments. **p < 0.01 vs. Ect1/E6E7

To investigate the potential involvement of miRNAs in CC development, differentially expressed miRNAs were analyzed through retrieving the gene expression datasets GSE55940. A total of 57 miRNAs were found to be differentially expressed (35 upregulated and 22 downregulated) in the CC tissues compared to matched tumor-adjacent tissues (Fig. 1a, b). Among these miRNAs, 8 miRNAs, miR-103a, miR-182, miR-21, miR-146a, miR-196a, miR-218, miR-214 and miR-7, were selected for further validation based on the fact that they have been previously identified in CC [16–21]. Among these eight miRNAs, 5 miRNAs, miR-103a, miR-182, miR-21, miR-146a and miR-196a, were found significantly upregulated, and 3 miRNAs, miR-218, miR-214 and miR-7 were significantly downregulated in tumor tissues compared with that in matched tumor-adjacent tissues (Fig. 1c). It is noticeable that miR-103a-3p emerged as the most significantly different miRNA between CC tissues and matched tumor-adjacent tissues. In addition, miR-103a-3p has been reported as an oncogene in several other types of cancers, such as colorectal cancer, thyroid cancer and oral squamous cell carcinoma [22–24]. However, its roles in the progression of CC remain undisclosed. Therefore, we chose miR-103a-3p for further studies.

Subsequently, we verified the miR-103a-3p expression levels in 70 paired tumor tissues and matched tumor-adjacent tissues. As expected, miR-103a-3p was significantly increased in tumor tissues compared to matched tumor-adjacent tissues (Fig. 1d). We further evaluate the association between miR-103a-3p expression and survival outcomes among patients with CC. According to the relative expression levels of miR-103a-3p in 70 paired tumor and normal tissues, the patients were divided into two groups: a high miR-103a-3p group (miR-103a-3p expression above the median value, *n* = 34) and a low miR-103a-3p group (miR-103a-3p expression below the median value, *n* = 36). The analysis showed the patients with miR-103a-3p-low expression have longer overall survival (OS) rate than that in patients with miR-103a-3p-high expression (Fig. 1e). In addition, we also measured the expression levels of miR-103a-3p in CC cell lines. The results showed that miR-103a-3p was significantly increased in CC cell lines compared with that in normal ectocervical cell lines (Ect1/E6E7) (Fig. 1f). Collectively, these results suggest that miR-103a-3p may be involved in the progression of CC.

To further understand the potential roles of miR-103a-3p in CC development, the associations between miR-103a-3p and clinicopathological features of CC were determined. As shown in Table 1, we found that high expression of miR-103a-3p was closely associated with Histological grades, FIGO stage and distant metastasis. However, no significant correlation was observed between miR-103a-3p expression and other clinicopathologic variables, such as age, tumor size and HPV infection. Collectively, these data suggest that miR-103a-3p may act as an effective biomarker for the prognosis of patients with CC.

### Knockdown of miR-103a-3p suppressed the cell proliferation and induced cell apoptosis

**Fig. 2:**
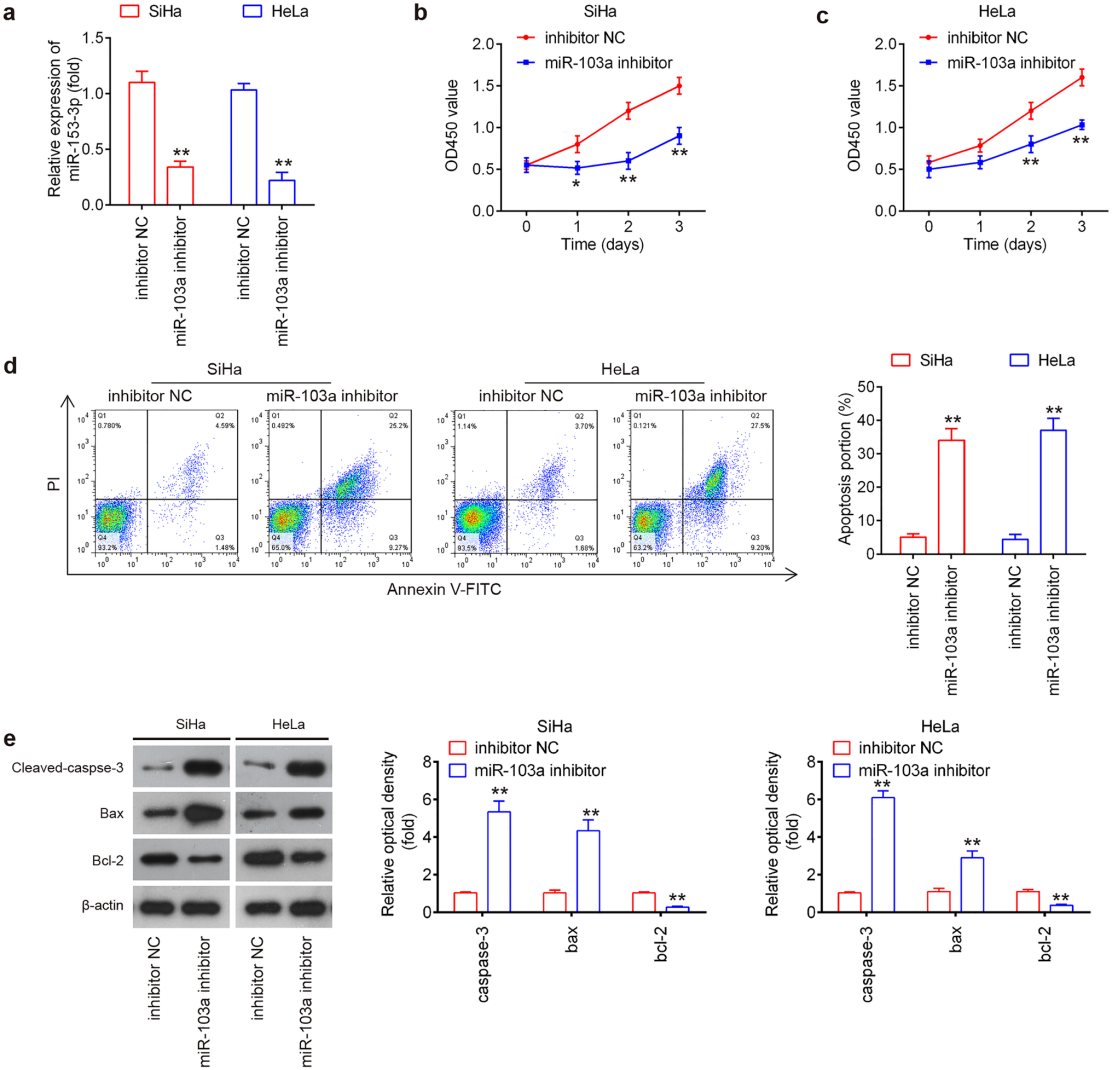
Knockdown of miR-103a-3p suppressed the cell proliferation and induced cell apoptosis. SiHa and Hela cells were transfected with the miR-103-3p inhibitor or inhibitor NC for 48 h, and the cells were used for analysis. **a** Transfection efficiency was assessed by reverse transcription-quantitative polymerase chain reaction analysis (*n* = 3). **b, c** Cell proliferation was measured using a Cell Counting Kit-8 assay at the indicated times (*n* = 3). **d** Apoptosis was detected by flow cytometry (*n* = 3). **e** Protein expression of cleaved caspase 3, Bcl-2 and Bax was measured by western blot analysis (*n* = 3). Data represent the mean ± SD of three independent experiments. *p < 0.05, **p < 0.01 vs. inhibitor NC group

The significant induction of miR-103a-3p expression in CC tissues prompted us to explore its possible functions in tumorigenesis of CC. miR-103-3p inhibitor or inhibitor NC were transfected into SiHa and Hela cells, which displayed the highest level of miR-103a-3p among four CC cell lines. As shown in Fig. 2a, miR-103-3p was notably downregulated in SiHa and Hela cells after transfection. CCK-8 assay analysis showed that miR-103a-3p knockdown significantly suppressed the cell proliferation compared to the NC control group (Fig. 2b, c). To examine whether the miR-103a-3p knockdown induced cell growth inhibition was associated with cell apoptosis, flow cytometry was performed to evaluate cell apoptosis. The results revealed that the apoptosis was significantly increased by miR-103-3p knockdown, compared with inhibitor NC transfected cells (Fig. 2d). Furthermore, western blot also showed that the expression of apoptosis-associated proteins, cleaved caspase 3 and Bax were markedly increased, while Bcl-2 was significantly decreased in miR-103-3p inhibitor group, compared with inhibitor NC group (Fig. 2e). These results pointed out that miR-103a-3p might decrease cell viability through inducing cell apoptosis.

### FBXW7 is a direct target of miR-103a-3p

**Fig. 3:**
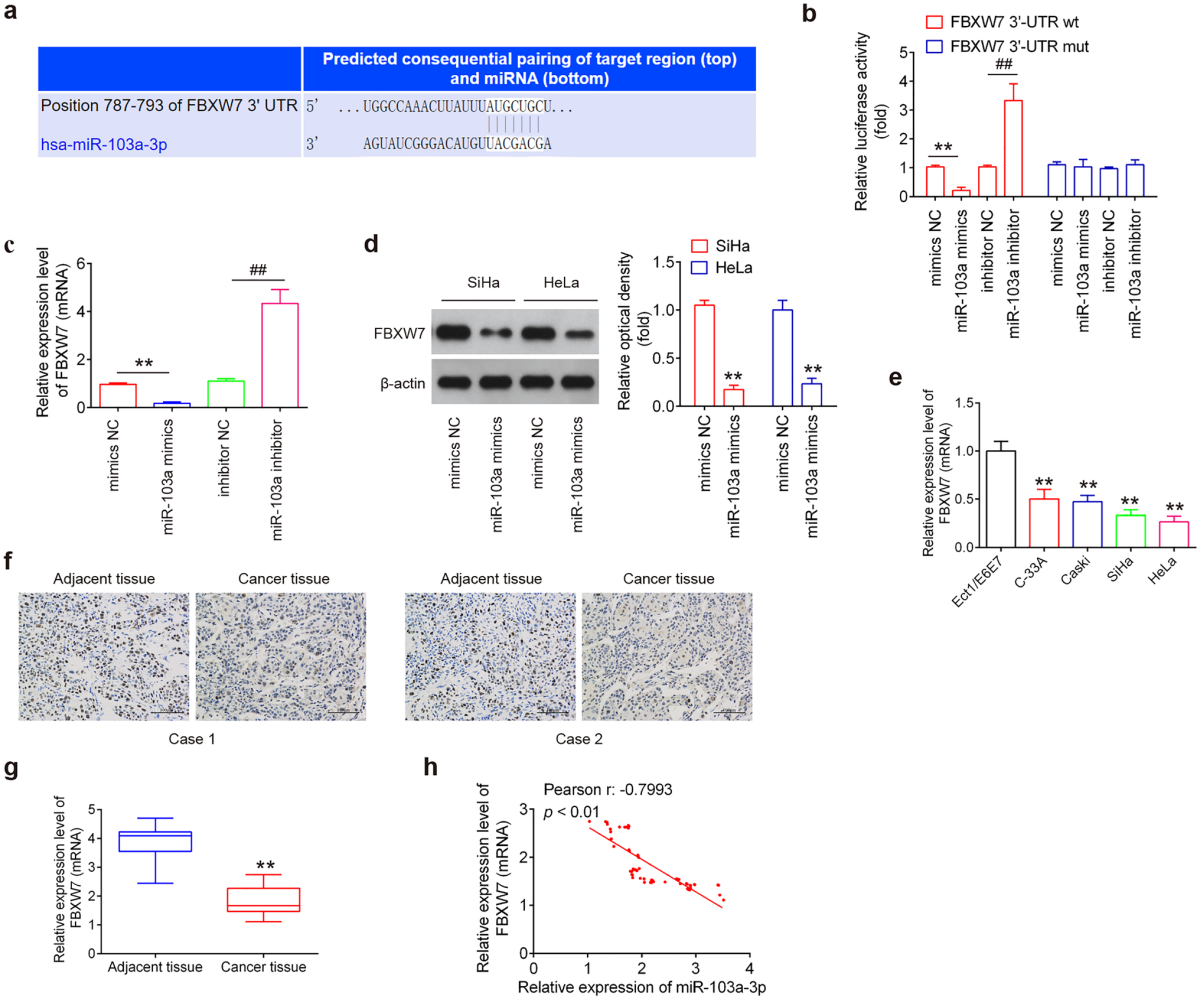
FBXW7 is a direct target of miR-103a-3p.** a** Putative binding sites of miR-103a-3p and FBXW7. **b** The relative luciferase activity of FBXW7 wild type (wt) or mutant (mut) 3’-UTR in 293 T cells following transfection with miR-103a-3p mimics, mimics NC, miR-103a-3p inhibitor or inhibitor NC, as indicated (*n* = 3). Data are presented as the mean ± standard deviation of three independent experiments. **p < 0.01 vs. mimics NC, ##p < 0.01 vs. inhibitor NC. **c, d** mRNA and protein expression of FBXW7 following transfection with miR-103a-3p mimics, mimics NC, miR-103a-3p inhibitor or inhibitor NC was measured by qRT-PCR and western blot analysis (*n* = 3). **p < 0.01 vs. mimics NC, ##*p* < 0.01 vs. inhibitor NC. **e** Expression was of FBXW7 examined in four CC cell lines and Ect1/E6E7 cells, used as a control, by qRT-PCR analysis (*n* = 3). Data are presented as the mean ± standard deviation of three independent experiments. **p < 0.01 vs. Ect1/E6E7.** f** Expression of FBXW7 was assessed by immunohistochemistry in CC tissues and matched tumor-adjacent tissues (magnification, × 200). **g** Expression of FBXW7 was measured by qRT-PCR analysis in CC tissues and matched tumor-adjacent tissues (*n* = 70). **p < 0.01 vs. adjacent tissues. **h** Pearson correlation analysis revealed a negative correlation between the expression of FBXW7 and miR-103a-3p (*r* = − 0.7993, *p* < 0.01)

To elucidate the molecular mechanism by which miR-103a-3p suppressed tumorigenesis, the Targetscan 7.0 (http://www.targetscan.org) and miRanda (http://www.microRNA.org) were performed to predict the target genes of miR-103a-3p. As shown in Fig. 3a, one potential binding site for miR-103a-3p was found in the 3’-UTR regions of FBXW7 mRNA. We next performed a luciferase reporter assay to confirm that FBXW7 was directly targeted by miR-103a-3p. The results showed that the miR-103a-3p overexpression significantly inhibited the luciferase activity combined with wild-type FBXW7-3’-UTR wild-type (wt) reporter, while the miR-103a-3p knockdown caused an increase in luciferase activity; however, no significant changes were observed using the FBXW7 3’-UTR mutant (mut) reporter with miR-103a-3p mimics or inhibitor (Fig. 3b). qRT-PCR and Western blot analysis demonstrated that miR-103a-3p overexpression significantly inhibited FBXW7 expression in SiHa and Hela cells at mRNA and protein level (Fig. 3c, d). We also analyzed the expressions of FBXW7 mRNA in CC cell lines by qRT-PCR. As shown in Fig. 3e, the expression of FBXW7 was significantly downregulated in CC cell lines compared to Ect1/E6E7. In addition, IHC was performed to examine the expression levels of FBXW7 in two paired CC tissues and adjacent tissues. It was also observed the significant reduction of FBXW7 expression in CC tissues compared to adjacent tissues (Fig. 3f). Moreover, we demonstrated that FBXW7 was significantly decreased in tumor tissues compared to matched tumor-adjacent tissues, and an inverse relationship was observed between miR-103a-3p and FBXW7 expression levels in tumor tissues (Fig. 3g, h). All these data suggest that FBXW7 is a functional target of miR-103a-3p, and FBXW7 down-expression in CC tumor tissues may be caused by upregulation of miR-103a-3p.

### FBXW7 overexpression suppressed the cell proliferation and induced cell apoptosis

**Fig. 4:**
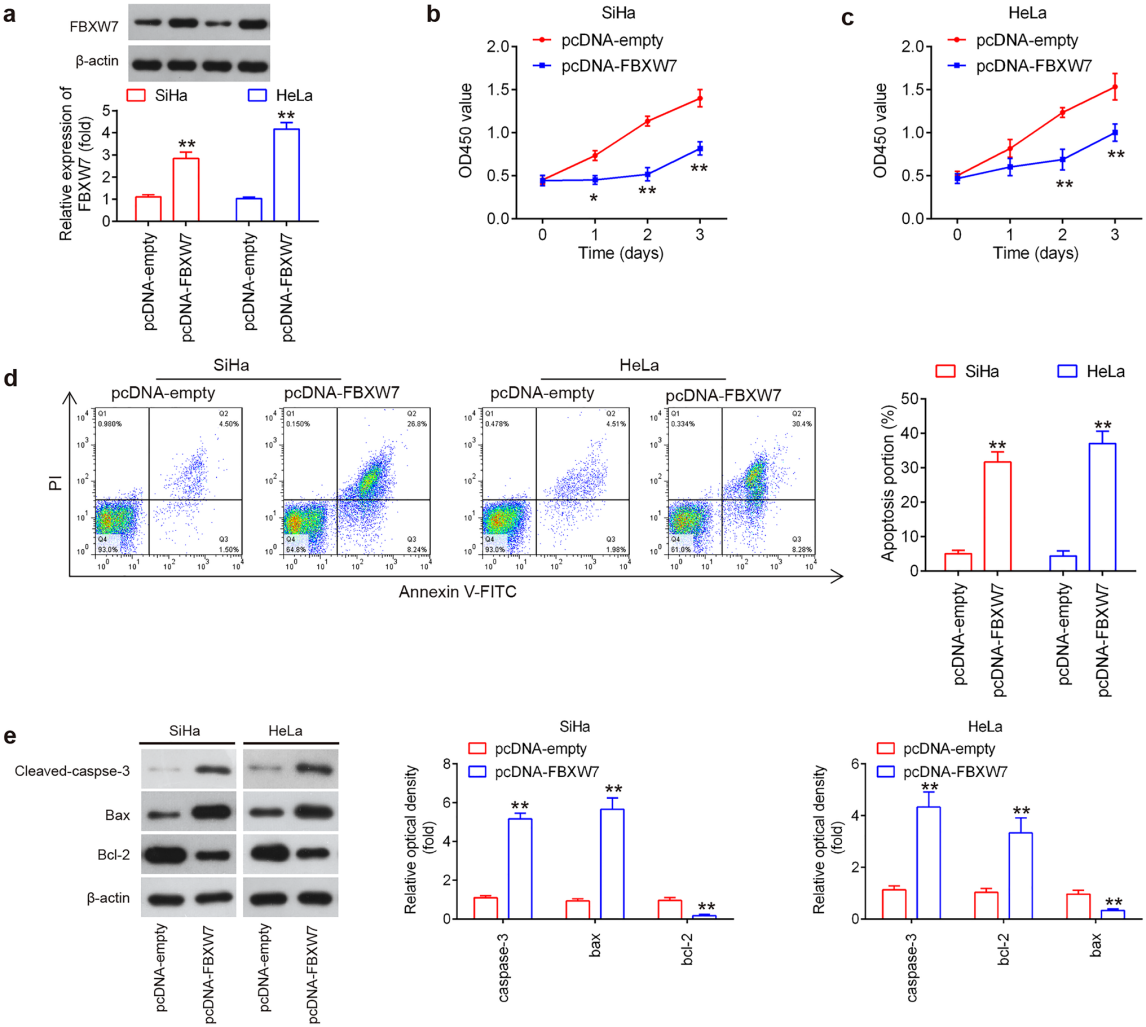
FBXW7 overexpression suppressed the cell proliferation and induced cell apoptosis. SiHa and Hela cells were transfected with the pcDNA-FBXW7 or pcDNA empty for 48 h, and the cells were used for analysis. **a** Transfection efficiency was assessed by western blot analysis (*n* = 3). **b, c** Cell proliferation was measured using a Cell Counting Kit-8 assay at the indicated times (*n* = 3). **d** Apoptosis was detected by flow cytometry (*n* = 3). **e** Protein expression of cleaved caspase 3, Bcl-2 and Bax was measured by western blot analysis (*n* = 3). Data represent the mean ± SD of three independent experiments. *p < 0.05, **p < 0.01 vs. pcDNA empty group

Previous evidence has shown that FBXW7 functions as a tumor suppressor in different human cancers, including non-small cell lung cancer (NSCLC) and renal cancer [25, 26]. Thus, we hypothesized that FBXW7 may act as a tumor-suppressive gene in CC. To this end, we transfected pcDNA-FBXW7 into SiHa and Hela cell lines. As shown in Fig. 4a, the expression levels of FBXW7 protein were notably upregulated after transfection with the pcDNA-FBXW7 in SiHa and Hela cells. Compared with that of control cells, overexpression of FBXW7 significantly decreased the growth rate of both CC cells (Fig. 4b, c), and a dramatically increased apoptosis was also observed in both CC cells (Fig. 4d). In addition, western blot analyses revealed that cleaved caspase 3 and Bax expression levels were markedly increased, while Bcl-2 was significantly decreased in pcDNA-FBXW7 transfected SiHa and Hela cells, compared with pcDNA empty transfected cells (Fig. 4e). All these results suggest that upregulation of FBXW7 suppressed the proliferation and promoted apoptosis of CC cells.

### miR-103a-3p inhibits cell proliferation and induces cell apoptosis by targeting FBXW7

**Fig. 5:**
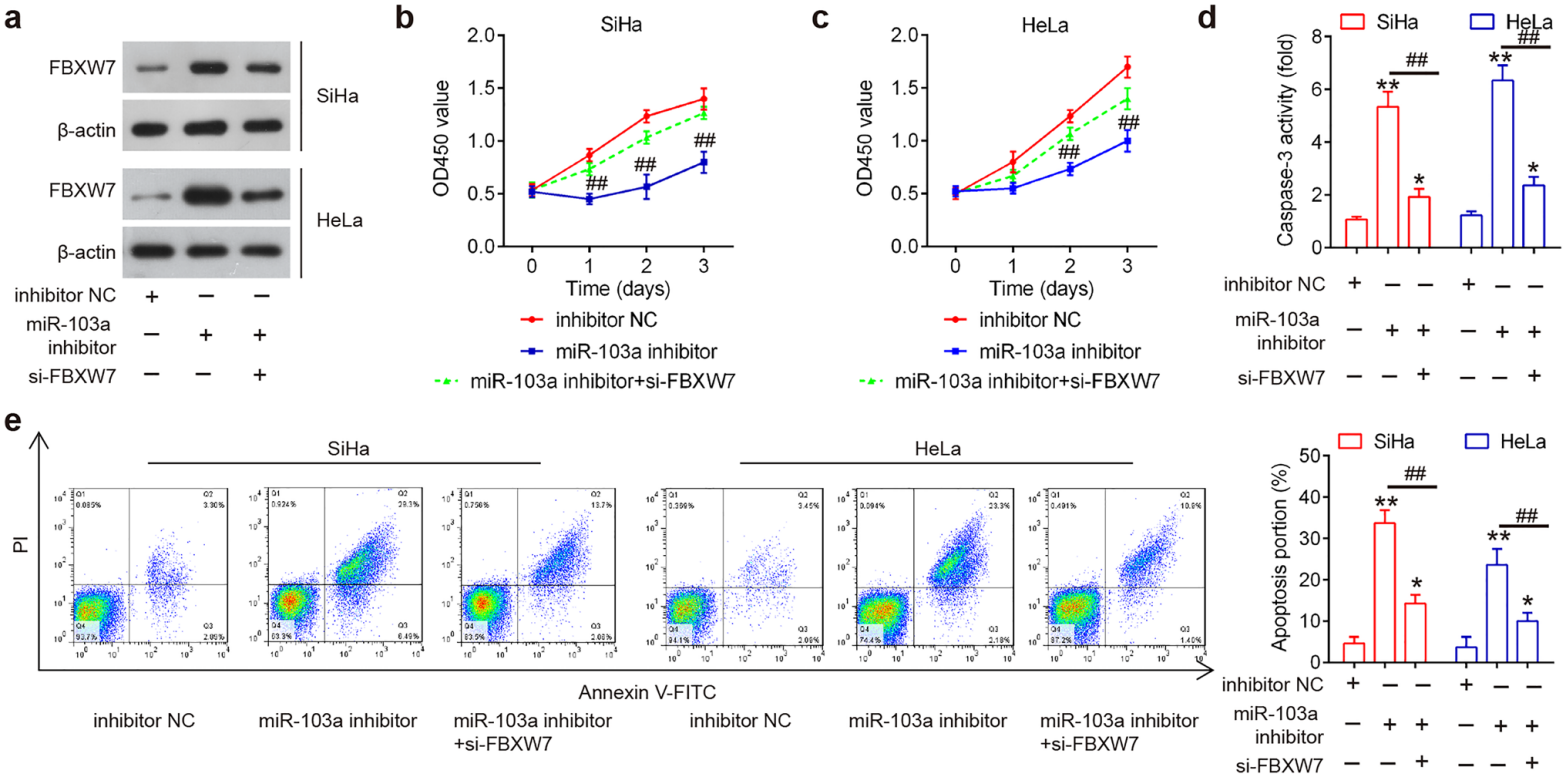
miR-103a-3p inhibits cell proliferation and induces cell apoptosis by targeting FBXW7. si-FBXW7 and miR-103a-3p inhibitor were co-transfected into SiHa and Hela cells for 48 h, and the cells were used for analysis. **a** Transfection efficiency was assessed by western blot analysis (*n* = 3). **b, c** Cell proliferation was measured using a Cell Counting Kit-8 assay at the indicated times (*n* = 3). **d** Caspase-3 activity was detected by a commercial kit (*n* = 3). **e** Apoptosis was detected by flow cytometry (*n* = 3). Data represent the mean ± SD of three independent experiments. *p < 0.05, **p < 0.01 vs. inhibitor NC group, ##*p* < 0.01 vs. miR-103a-3p inhibitor

To explore whether FBXW7 mediates tumor-suppressive effects of miR-103a-3p knockdown on CC cells, si-FBXW7 and miR-103a-3p inhibitor were co-transfected into SiHa and Hela cells. As shown in Fig. 5a, FBXW7 expression was significantly decreased in SiHa and Hela cells after si-FBXW7 transfection. As shown in Fig. 5b, c, the inhibitory effects of miR-103a-3p knockdown on the cell proliferation was attenuated by FBXW7 inhibition. In addition, the increased caspase-3 activity caused by miR-103a-3p inhibitor was also reduced by FBXW7 inhibition (Fig. 5d). Meanwhile, FBXW7 inhibition significantly reduced the increased apoptosis portion caused by miR-103a-3p knockdown (Fig. 5e). These findings suggested that miR-103a-3p inhibition suppressed the proliferation and promoted cell apoptosis via regulating FBXW7.

### miR-103a-3p influences FBXW7 mediated the degradation of the most cancer-relevant substrates

**Fig. 6:**
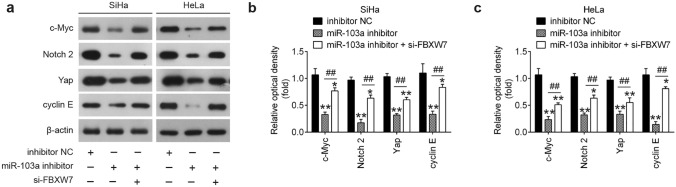
miR-103a-3p knockdown suppressed the oncoproteins expressions by targetingFBXW7. si-FBXW7 and miR-103a-3p inhibitor were co-transfected into SiHa and Hela cells for 48 h, and the cells were used for analysis. **a** The expressions of cyclin E, c-Myc, Yap and Notch2 proteins were determined in SiHa and Hela cells by western blot (*n* = 3). **b, c** Protein bands were analyzed semi-quantitatively using ImageJ software, normalized to β-actin density. Data represent the mean ± SD of three independent experiments. *p < 0.05, **p < 0.01 vs. inhibitor NC group, ##*p* < 0.01 vs. miR-103a-3p inhibitor

FBXW7, a well-known tumor suppressor, has been demonstrated to control the degradation of some oncoproteins such as cyclin E, c-Myc, Yap and Notch2 [27–31]. Therefore, we hypothesized that by targeting FBXW7, miR-103a-3p might affect these oncoproteins expressions in CC cells. cyclin E, c-Myc, Yap and Notch2 protein levels were determined in SiHa and Hela cells after transfection with the miR-130a-3p inhibitor or si-FBXW7. As shown in Fig. 6a–c, miR-103a-3p knockdown significantly suppressed the expression levels of cyclin E, c-Myc, Yap and Notch2 compared with those in inhibitor NC group. In contrast, siRNA interference of FBXW7 partially reversed the inhibitory effects of miR-103a-3p inhibitor on the expressions of these oncoproteins. Taken together, these results suggest that miR-103a-3p may exert oncogene role in CC cells by repressing FBXW7, and thus indirectly regulating these oncoproteins.

## Discussion

In this study, we found that miR-103a-3p expression levels were upregulated in CC tissues and cell lines. Downregulation of the miR-103a-3p significantly suppressed cell proliferation and promoted cell apoptosis in vitro. Furthermore, FBXW7 was identified as a functional target of miR-103a-3p, and miR-103a-3p knockdown increased the FBXW7 expression, which in turn suppressed the oncoproteins expressions, and thus inhibited malignancy in CC cells.

Increasing evidence has demonstrated that miRNAs are differently expressed in CC tissues, and play important roles in the development and progression of CC [7, 32, 33]. For example, Shi et al. [34] have demonstrated that miR-362 upregulation repressed cell migration and invasion through targeting SIX1. Chen et al. [35] have reported that miR-499a targeted SRY-box transcription factor 6 to promote the proliferation of CC cells. Yuan et al. [36] showed that miR-138 upregulation suppressed CC cell growth in vivo. In this study, the differentially expressed miRNAs that were screened based on GSE55940 microarray data retrieved from Gene Expression Omnibus (GEO, miR-103a-3p was identified as one of the most upregulated miRNAs in CC tissues. Of great interest, this miRNA has been recently discovered and it functions as an oncogene in several types of human cancers [37–39]. However, the expression pattern and role of miR-103a-3p in CC remain unknown. In this study, miR-103a-3p was significantly upregulated in CC tissues and CC cell lines, and high expression of miR-103a-3p was closely associated with histological grades, FIGO stage and distant metastasis. We also found that CC patients with high expression of miR-103a-3p exhibited poor OS rate. As we known, chemoresistance is the main obstacle in successfully treating CC and indicates poor prognosis of patients with CC. A recent study reported that miR-103a-3p potentiated chemoresistance in non-small cell lung carcinoma [40]. The miR-103a-3p associated poor overall survival might be caused by chemoresistance.

Recent studies reported that miR-103a-3p functions as an oncogene with functional effects on the proliferation and apoptosis in diverse caners. For instance, Hu et al. [37] demonstrated that miR-103a-3p overexpression caused cell proliferation promotion in gastric cancer (GC). Xia et al. [38] found that miR-103a-3p promoted hepatocellular carcinoma growth by targeting AKAP12. In addition, in colorectal cancer cells, miR-103a-3p was demonstrated to promote cell proliferation and migration by targeting DICER and PTEN [39]. However, certain studies have observed that the function of miR-103a-3p 7 could be characterized as a tumor suppressor gene in several human cancers. In a study investigating the impact of miR-103a-3p on prostate cancer (PCa) cells, enhancement of miR-103a-3p inhibited migration and invasion of PCa cells by regulating the expression of the oncogenic tumor protein D52 (TPD52) [41]. Moreover, miR-103a-3p inhibited malignant progression of glioma by binding to the FEZF1 3’-UTR [42]. miR-103a-3p was also found to be lowly expressed in bladder cancer and acted as a tumor suppressor by targeting circTCF25 [43]. These conflicting studies indicated that miR-103a-3p expression has tissue specificity and may serve as a potential diagnostic and prognosis marker for these human cancers. In this study, we demonstrated that miR-103a-3p knockdown inhibited cell proliferation, and promoted the apoptosis of SiHa and Hela cells, indicating that miR-103a-3p may function as an oncogene in CC.

Several direct targets have been identified, including AKAP12 in hepatocellular carcinoma, PTEN in non-small-cell lung cancer and D52 in prostate cancer, have been reported to be targets of miR-103a-3p [38, 41, 44]. In our current study, FBXW7 was validated as a direct and functional target of miR-103a-3p in cervical cancer. FBXW7, one of the F-box protein family members, function as a tumor-suppressing gene in various human cancers [45, 46]. For example, Li et al. [47] showed that FBXW7 overexpression inhibited gastric cancer (GC) progression by inducing apoptosis and growth arrest. Cheng et al. [48] found that FBXW7 inhibited the migration and invasion in melanoma cells. Moreover, FBXW7 mutation could be able to promote cell migration and invasion of CC cells [49]. Interestingly, FBXW7 has recently been studied as a target gene of miRNAs in various cancers. For example, Liu et al. [50] showed that miR-223 could bind to the FBXW7 gene and inhibit its expression, ultimately increasing the proliferation and preventing the apoptosis of CRC cells through the Notch and Akt/mTOR signaling pathways. Da et al. [51] reported that miR-92a promoted NSCLC cell growth by targeting tumor suppressor gene FBXW7. In our study, FBXW7 was confirmed to be a target of miR-103a-3p. What is more, FBXW7 was found to be significantly decreased in CC tissues, and inversely correlated with miR-103a-3p in CC tissues. Furthermore, FBXW7 overexpression mimicked the effects of miR-103a-3p knockdown in CC cells, and its knockdown reversed the inhibitory effect of miR-103a-3p inhibition in CC cells. It is well known that the tumor suppressor functions of FBXW7 attributed to its proteolytic regulation of oncogenic substrates such as cyclin E, Yap, c-Myc and Notch, and likely unknown substrates as well [52–55]. In our study, miR-103a-3p knockdown led in a strong reduction of c-Myc, Notch2 and cyclin E through upregulating FBXW7 in CC cells. All the above studies have shown that miR-103a-3p inhibition promoted FBXW7 expression, subsequently inhibited these oncogenic substrates; thus, suppressing proliferation and enhancing apoptosis in CC cells.

In recent years, Hippo signaling exerts a critical role in modulating cell proliferation and has been demonstrated to be involved in the oncogenesis of a variety of tumors including CC [56–58]. For example, Hippo pathway activation was found to encourage proliferation and migration of cervical cancer cells through a positive signaling loop involving the epidermal growth factor receptor (EGFR) [59]. Another study showed that Ajuba LIM Protein (AJUBA) negatively regulated the Hippo signaling pathway and increased the resistance of cervical cancer cells to cisplatin, which is also associated with reduced survival time [60]. Notably, research reports on the regulation of the Hippo pathway by miRNAs, such as miR-135b and miR-31 have gradually attracted attention [61, 62]. A recent study reported that miR-103a-3p promoted the activation of Hippo pathway via targeting large tumor suppressor kinase 2 (LAST2), ultimately promoting hepatoma cell metastasis and EMT [63]. Another study also showed that miR-103a-3p knockdown suppressed HIF1A expression by targeting the core molecules LATS2 and SAV1 of the Hippo/YAP1 pathway, contributing to reduced proliferation, invasion, migration, glycolysis and angiogenesis in colorectal cancer [22]. Therefore, it is plausible to consider that miR-103a-3p has a similar action mechanism in CC via regulating Hippo signaling pathway. However, the regulatory mechanism of miR-103a-3p involving Hippo pathway is not analyzed in this study. It waits for more efforts to focus on the action mechanisms of miR-103a-3p in the incoming studies.

Although some interesting results were found in the present study, there were still some limitations. First, the sample size of CC patients is not big enough. Large part of this data was engaged in the molecular aspects but this is excruciatingly insufficient to reach the clinical conclusion. More samples and further studies are needed to disclosure the relationship between miR-103a-3p and the clinicopathological features in cervical cancer. Secondly, besides FBXW7, other downstream targets such as AKAP12 [38], PTEN [64] and D52 [41] have been reported to be targets of miR-103a-3p. We believe that some of these genes may play important roles in CC, which need to be further explored. Thirdly, a previous study reported that miR-103a-3p potentiated chemoresistance in non-small cell lung carcinoma [40]. Therefore, the miR-103a-3p associated poor overall survival might be caused by chemoresistance, which needs further research. Finally, we will investigate the levels and function of miR-103a-3p in other human cancers in future.

In conclusion, our findings demonstrated that miR-103a-3p functions an oncogenic role in cervical cancer by inhibiting FBXW7 function. Our findings indicated that the miR-103a-3p/FBXW7 axis may serve as a potential biomarker or new target in CC therapy.

## Data Availability

All data generated and/or analyzed during the present study are included in this article.
